# Differential Expression of MicroRNAs in Leprosy Skin Lesions

**DOI:** 10.3389/fimmu.2017.01035

**Published:** 2017-08-25

**Authors:** Cleverson T. Soares, Ana P. F. Trombone, Luciana R. V. Fachin, Patricia S. Rosa, Cássio C. Ghidella, Rodrigo F. Ramalho, Mabel G. Pinilla, Alex F. Carvalho, Dirce N. Carrara, Fernando A. Soares, Andrea F. F. Belone

**Affiliations:** ^1^Department of Anatomic Pathology, Instituto Lauro de Souza Lima, São Paulo, Brazil; ^2^Department of Health Science, Universidade do Sagrado Coração, São Paulo, Brazil; ^3^Division of Research and Education, Instituto Lauro de Souza Lima, São Paulo, Brazil; ^4^Ambulatory of Leprosy, Jardim Guanabara Health Center, Rondonópolis, Brazil; ^5^Laboratory of Genomics and Molecular Biology, CIPE, A.C. Camargo Cancer Center, São Paulo, Brazil; ^6^Department of Medical Technology, School of Medicine, University of Concepción, Concepción, Chile; ^7^Department of Preventive Veterinary Medicine, Federal University of Minas Gerais, Belo Horizonte, Brazil; ^8^Department of Anatomic Pathology, A.C. Camargo Cancer Center, São Paulo, Brazil

**Keywords:** leprosy, expression, microarray, microRNA, signaling pathways

## Abstract

Leprosy, a chronic infectious disease caused by *Mycobacterium leprae*, is a major public health problem in poor and developing countries of the Americas, Africa, and Asia. MicroRNAs (miRNAs), which are small non-coding RNAs (18–24 nucleotides), play an important role in regulating cell and tissue homeostasis through translational downregulation of messenger RNAs (mRNAs). Deregulation of miRNA expression is important for the pathogenesis of various neoplastic and non-neoplastic diseases and has been the focus of many publications; however, studies on the expression of miRNAs in leprosy are rare. Herein, an extensive evaluation of differentially expressed miRNAs was performed on leprosy skin lesions using microarrays. Leprosy patients, classified according to Ridley and Jopling’s classification or reactional states (R1 and R2), and healthy controls (HCs) were included. Punch biopsies were collected from the borders of leprosy lesions (10 tuberculoid, 10 borderline tuberculoid, 10 borderline borderline, 10 borderline lepromatous, 4 lepromatous, 14 R1, and 9 R2) and from 9 HCs. miRNA expression profiles were obtained using the Agilent Microarray platform with miRBase, which consists of 1,368 *Homo sapiens* (hsa)-miRNA candidates. TaqMan quantitative real-time reverse transcription polymerase chain reaction (RT-PCR) was used to validate differentially expressed miRNAs. Sixty-four differentially expressed miRNAs, including 50 upregulated and 14 downregulated (fold change ≥2.0, *p*-value ≤ 0.05) were identified after comparing samples from patients to those of controls. Twenty differentially expressed miRNAs were identified exclusively in the reactional samples (14 type 1 and 6 type 2). Eight miRNAs were validated by RT-PCR, including seven upregulated (hsa-miR-142-3p, hsa-miR-142-5p, hsa-miR-146b-5p, hsa-miR-342-3p, hsa-miR-361-3p, hsa-miR-3653, and hsa-miR-484) and one downregulated (hsa-miR-1290). These miRNAs were differentially expressed in leprosy and several other diseases, especially those related to the immune response. Moreover, the integration of analysis of validated mi/mRNAs obtained from the same samples allowed target pairs opposite expression pattern of hsa-miRNA-142-3p and AKR1B10, hsa-miRNA-342-3p and FAM180b, and hsa-miRNA-484 and FASN. This study identified several miRNAs that might play an important role in the molecular pathogenesis of the disease. Moreover, these deregulated miRNAs and their respective signaling pathways might be useful as therapeutic markers, therapeutic targets, which could help in the development of drugs to treat leprosy.

## Introduction

Leprosy, a chronic infectious disease caused by *Mycobacterium leprae*, is a major public health problem in poor and developing countries in the Americas, Africa, and Asia (1). Sporadic cases have been reported in developed countries, but usually in immigrants from endemic areas (2). *M. leprae* is an obligate intracellular parasite with tropism for the peripheral nervous system, and thus neural involvement is a feature of all forms of leprosy. The bacillus has slow replication, a long incubation period, and few genes controlling its metabolism (3). As a consequence, the disease evolves slowly for years or even decades, resulting in different clinical presentations and mimicking numerous other diseases; thus, treating this disease is very challenging. Leprosy is classified according to Ridley and Jopling (R&J), using two polar forms, tuberculoid (TT) and lepromatous (LL), and an intermediate group that is subdivided into borderline tuberculoid (BT), borderline borderline (BB), and borderline lepromatous (BL) (4). During the course of the disease, reactional episodes can occur, some of which are intense and destructive (5).

There are basically two types of reactions in leprosy. A type 1 reaction (R1) occurs with relative preservation of specific cellular immunity against *M. leprae*. A type 2 reaction (R2) occurs with poor preservation or absence of cellular immunity. R1 is an immunocellular response that occurs mainly in patients with TT and the borderline forms of the disease (BT, BB, and BL). The clinical signs of R1 include swelling and erythema of preexisting lesions, and when very intense, there can be necrosis and ulceration. Histopathological examination typically reveals more extensive, confluent, and poorly delimited granulomas associated with interstitial and intracellular edema, fibrin deposition, focal or confluent necrosis, and varying degrees of epithelial hyperplasia (5). R2 is clinically and histopathologically different from R1; it occurs in individuals with the lepromatous forms of leprosy (BL and LL) and is clinically characterized by erythematous papules, nodules, or plaques on specific lesions (usually during regression), and depending on the intensity of the reaction can evolve with suppuration, necrosis, and ulceration. The cutaneous lesions are accompanied by general systemic manifestations such as fever, myalgia, asthenia, inappetence, and often inflammatory manifestations in all parts of the body that contain bacillary antigens. In addition, neuritis, arthritis, painful lymphadenopathy, buccopharyngeal lesions, laryngitis, hepatomegaly and splenomegaly, bone lesions, iridocyclitis, uveitis, orchitis, and glomerulitis associated with proteinuria, and hematuria can occur. At any of these locations, the histological presentation is characterized by an acute or subacute non-granulomatous inflammatory reaction with vascular proliferation, endothelial swelling, venocapillary thrombi, serous–fibrin–neutrophil exudation, disorganization of preexisting granulomas, and the formation of microabscesses (5).

There is no specific treatment to prevent the occurrence of these reactional phenomena and no blood marker that identifies reactional episodes or their intensity. In addition, there is no any effective treatment regimen for all cases. During episodes, neurological lesions often worsen, typically resulting from direct aggravation of the neural branches and other tissues, which can lead to permanent functional disabilities.

The pathophysiological mechanisms of leprosy progression and reactional episode onset are virtually unknown (5). It is also not known how the bacillus, initially present in only a few lesions and predominantly in the peripheral neural branches (indeterminate form), is able to spread throughout the entire body, parasitizing different types of cells such as macrophages, Schwann cells, smooth muscle cells, endothelial cells, fibroblasts, melanocytes, and potentially even epithelial cells. It is likely that *M. leprae* is able to subvert host immune mechanisms by modifying the expression of genes in parasitized cells, making these cells an environment conducive to the survival of the bacilli; in addition, it is speculated that a similar strategy is used for their spread to adjacent tissues and distant organs.

There are a large number of studies showing that non-coding RNAs are important for the maintenance of cell and tissue homeostasis and that their dysregulation is involved in the development of diseases (6). Among these non-coding RNAs, microRNAs (miRNAs) play an important role in the downregulation of gene expression at the translational level, through specific binding to messenger RNA (mRNA), which results in translational repression and/or degradation of target mRNA (7).

It is well known that altered expression of miRNAs occurs in various types of diseases, but most often this has been described in neoplasms. Studies have provided a better understanding of the pathophysiological mechanisms of different diseases at the molecular level, some of which have displayed peculiar miRNA expression patterns allowing for their molecular classification (8). The miRNAs that are differentially expressed in these diseases have also been the subject of studies to discover new biomarkers with prognostic, predictive, or therapeutic potential (9). However, there have been few studies related to the expression of miRNAs in infectious diseases, particularly with respect to leprosy (10, 11).

In a recent study on the expression of mRNAs in skin samples using microarrays, hundreds of differentially expressed mRNAs were identified in the spectrum of leprosy and its reactional states suggesting their participation in the pathophysiology of these conditions (12). Some of these differentially expressed mRNAs might be regulated in cells that comprise the granulomas of the leprosy lesions.

Leprosy is a disease that is difficult to treat, and this is especially true of the reactional episodes. Drugs currently used, including corticosteroids and thalidomide, significantly disrupt homeostasis, leading to difficult-to-control disorders such as obesity, diabetes, immunodeficiency, and teratogenesis, among others (13). Thus, this disease is challenging in all aspects, and there is an urgent need for new drugs to treat reactional episodes; it is also important to discover markers that can predict or identify these reactional states (14). miRNAs play an important role in triggering and maintaining many diseases, and therefore they could be important in the pathophysiology of leprosy. Thus, this study sought to evaluate the expression of miRNAs in the skin lesions of patients comprising the entire spectrum of leprosy.

This study utilized microarrays to identify differentially expressed miRNAs that might be involved in the pathophysiology of leprosy. These candidate miRNAs were hypothesized to represent novel markers and therapeutic targets for leprosy and its reactional states, which will be the focus of future research.

## Materials and Methods

### Project Design, Sample Collection, and Classification

The events in this study occurred in the following order: patients who were consulted at the leprosy Outpatient Clinics of the Lauro de Souza Lima Institute (ILSL—Bauru, São Paulo) and Rondonópolis (Mato Grosso) were examined by leprologists and underwent two skin biopsy procedures. One biopsy was processed by histopathological analysis and bacilloscopy. The other was stored immediately after collection in RNAlater solution for future extraction of RNA. This study was performed using the same RNA material extracted from samples used in a recent publication reporting mRNA expression in leprosy (12).

After clinical and histopathological assessments and bacilloscopy, the patients were classified according to Ridley and Jopling’s criteria of disease and reactions (TT, BT, BB, BL, LL, R1, and R2) (4, 5). Sixty-seven samples of leprosy lesions (TT = 10, BT = 10, BB = 10, BL = 10, LL = 4, R1 = 14, and R2 = 10), and nine skin biopsies from healthy subjects used as controls (healthy control; HC = 9), were collected. To avoid large variations in histological patterns that could interfere with miRNA expression, only samples from lesions on the trunk and upper and lower limbs were used. No samples were collected from the scalp, face, palms, and soles.

The data for all patients, including age, gender, and ethnicity, as well as the identification of each sample, are listed in Table S1 in Supplementary Material. It is important to mention that ethnic factors were not considered since previous studies demonstrated the high individual ancestral variability was observed in Brazilian population (independently of different geographical regions) which reflects a singular proportion of Amerindian, European, and African ancestries in its mosaic genome, and that in this population it is not possible to predict the color of persons from their genomic ancestry nor the opposite. The classic skin color stratification for additional analysis is not useful in the population investigated (15). This study was approved by the Research Ethics Committees of Hospital A.C. Camargo (no. 1535/11) and the Instituto Lauro de Souza Lima (ILSL—no. 033/2011).

The following comparisons were used to identify differentially expressed miRNAs in terms of the leprosy spectrum and different reactional states: (1) disease (TT + BT + BB + BL + LL + R1 + R2) vs. HC; (2) clinical forms (TT + BT + BB + BL + LL) vs. HC; (3) between polar forms (TT vs. LL); (4) tuberculoid (TT + BT) vs. HC; (5) lepromatous (BL + LL) vs. HC; (6) TT vs. HC; (7) LL vs. HC; (8) borderline leprosy (BT + BB + BL) vs. HC; (9) reactional type 1 (R1) vs. its respective clinical forms (TT + BT + BB + BL); (10) R1 vs. HC; (11) reactional type 2 (R2) vs. its respective clinical forms (BL + LL); (12) R2 vs. HC.

### Extraction and Analysis of RNA Quality and Integrity

Skin biopsy samples (stored in RNAlater) were individually fragmented using a scalpel and transferred to a ceramic bead tube (CK28-Bertin Technologies). QIAzol reagent (700 µL; Qiagen) was added, and the samples were processed (homogenization and lysis) in a Precellys 24 homogenizer (Bertin Technologies) with three cycles of 10-s pulses, and further incubated at 4°C for 5 min. Total RNA (including miRNA) was extracted using the miRNeasy Mini Kit (Qiagen) according to the manufacturer’s instructions in a QIAcube apparatus (Qiagen). The RNA was recovered in 30 µL of RNase-free water. RNA quantification was performed using a Nanodrop 2000 (ThermoScientific), and integrity was evaluated using a Bioanalyzer 2100 electrophoresis system (GE Healthcare Bio-Sciences). Samples with low quality or insufficient RNA for analysis were excluded.

### Labeling and Hybridization of miRNA

To evaluate the expression of miRNA, 300 ng of each total RNA sample was subjected to RNA (Cy3) labeling, performed according to the manufacturer’s instructions (miRNA microarray system—complete labeling and hyb kit; protocol: version 2.4, September 2011; Agilent Technologies). The slides used in these assays were miRNA—human miRNA microarray (G4872A-031181—8 × 60 K) (G&E Healthcare Bio-Sciences). The oligoarrays were hybridized with fluorescent targets at 55°C for 20 h in a hybridization oven. After hybridization, the slides were processed using buffers provided by the manufacturer (Agilent Technologies) to eliminate non-specific targets and background interference, and then were subjected to a drying process that included washing in acetonitrile for 1 min and in a washing and stabilization solution for microarrays for 1 min. The arrays were digitized using the Agilent Bundle Scanner (Agilent, USA) with a resolution of 3 µm and Feature Extraction software (Version 11.0). The digitized images of each array were submitted for data quality analysis using Agilent Genespring software version 11.0 (Agilent^®^).

### Selection of miRNAs for Reverse Transcription Polymerase Chain Reaction (RT-PCR) Validation

After analyzing differentially expressed miRNAs among diverse samples representing the leprosy spectrum and different reactional forms, 51 miRNAs were selected for reverse transcription polymerase chain reaction (RT-PCR) validation; some were highly upregulated or downregulated based on most comparisons, whereas others were expressed in either type 1 or type 2 reactional states. An unpaired asymptotic *T* test was employed with a Bonferroni FWER correction for statistical analysis, and those with fold change ≥2.0 and a *p*-value ≤ 0.05 were validated by RT-PCR.

### Validation of miRNAs by RT-PCR

Complementary DNA (cDNA) was synthesized from total RNA by adding 0.5 µg of oligo-dT15 to 2 µg of total RNA in a final volume of 5 µL, and incubating the sample at 70°C for 10 min and cooling on ice for oligo annealing. Reverse transcription was performed using SuperScript III enzyme (Invitrogen) according to the manufacturer’s instructions in a final volume of 20 µL. The sample was incubated at 50°C for 1 h, followed by 15 min at 70°C. Aliquots of the obtained cDNA were diluted 10-fold and stored at −20°C.

After selecting genes, custom PCR plates were ordered in a 96 × 1 format (Qiagen—miRNA #CMIHS02125). Twenty-four miRNA samples, representing the entire spectrum of disease, the reactional forms, and controls, were validated.

Complementary DNA synthesis from miRNA samples was performed using the miScript II RT Kit and HiSpec Buffer (Qiagen). The obtained cDNA was subjected to the RT-PCR protocol of the miScript miRNA PCR Array with the miScript SYBR Green PCR Kit (Qiagen), using an ABI VIIA 7 device (Applied Biosystems).

After completing the reaction, the quality of data was analyzed using SDS 2.3 software (Applied Biosystems). The dissociation curves were analyzed for amplification of genomic DNA, primer dimers, and splicing variants. Using amplification plots, the fluorescence intensity threshold was adjusted in the exponential phase of the graph in which the cycle threshold values of each reaction were considered. Duplicates with SDs of less than 0.5 were considered acceptable.

The relative expression in each group was compared using a non-parametric Mann–Whitney test.

### Filtering miRNA/mRNA Target Pairs Opposite Expression Pattern

We identify from our list of differentially expressed miRNAs and mRNAs those pairs that (i) were previously described as a validated interaction partners and (ii) show opposite expression pattern in our data. For this analysis, we consider only miRNA/mRNAs found as differentially expressed in each of the following group comparisons (Clinical forms vs. HC, TT vs. HC, LL vs. HC, R1 vs. HC and R2 vs. HC). The mRNAs were selected from a previous already published study by the same authors, in which the same set of samples used in this study were analyzed (12). Each group comparison was analyzed separately. Basically, the computational filtering strategy consisted in collecting from the MirWalk database (16) all validated mRNA targets for all differentially expressed miRNAs found in the comparisons mentioned earlier. After that, by using an in-house R script, we search for validated pairs with opposing expression pattern in our data, i.e., for each miRNA downregulated we searched for upregulated mRNA targets and for each miRNA upregulated we searched for downregulated mRNA in our data.

### Pathway Enrichment Analysis

The genes selected from the miRNA/mRNA analysis were submitted to pathway enrichment analysis by using the ReactomeFIViz plugin from the Cytoscape software^1^ that performs searches in the Reactome Database.^2^ The pathway enrichment analysis was applied separately to the gene sets selected from each group comparison (Clinical forms vs. HC, TT vs. HC, LL vs. HC, R1 vs. HC and R2 vs. HC) and also to a single gene set consisted of all selected genes found in these comparisons.

### Microarray Data Accession Number

The microarray data set has been submitted to the Gene Expression Omnibus database at NCBI^3^ and assigned accession number GSE102314.

## Results

### Differentially Expressed miRNAs in Disease and Reactional Episodes

Comparing disease (TT + BT + BB + BL + LL + R1 + R2) vs. HC groups, 64 miRNAs (50 upregulated and 14 downregulated) were differentially expressed (Table S2 in Supplementary Material; Table 1). Upon comparing clinical forms (TT + BT + BB + BL + LL) vs. HC groups, 20 miRNAs (14 upregulated and 6 downregulated) were differentially expressed (Table S3 in Supplementary Material; Table 2). Only one downregulated miRNA (hsa-miR-181a*) was differentially expressed between the polar forms (TT vs. LL) (Table S4 in Supplementary Material). Tables S5–S9 in Supplementary Material list all miRNAs differentially expressed when making the following comparisons: tuberculoid form (TT + BT) and HC (17 miRNAs—Table S5 in Supplementary Material), lepromatous form (BL + LL) and HC (18 miRNAs—Table S6 in Supplementary Material), TT and HC (17 miRNAs—Table S7 in Supplementary Material), LL and HC (18 miRNAs—Table S8 in Supplementary Material), and borderline leprosy (BT + BB + BL) and HC (18 miRNAs—Table S9 in Supplementary Material).

**Table 1:** The 10 most upregulated or downregulated microRNAs differentially expressed, based on microarray, in disease (TT + BT + BB + BL + LL + R1 + R2) vs. HC, fold change (FC) ≥ |2| and *p* ≤ 0.05.

Gene symbol	Regulation	FC microarray	*p* Value
hsa-miR-181a*	Up	35.59919	8.81E−12
hsa-miR-155	Up	26.33272	5.46E−19
hsa-miR-21*	Up	14.50879	1.88E−10
hsa-miR-3607-5p	Up	11.07103	6.08E−05
hsa-miR-3926	Up	11.01895	1.39E−05
hsa-miR-4261	Up	9.929722	1.51E−04
hsa-miR-378*	Up	9.214098	5.55E−05
hsa-miR-423-3p	Up	9.161276	9.57E−07
hsa-miR-501-3p	Up	9.157262	2.58E−05
hsa-miR-31	Up	9.013615	0.001011495
hsa-miR-141	Down	−2.25298	7.14E−06
hsa-miR-193b	Down	−2.35208	5.69E−09
hsa-miR-205	Down	−2.4154	2.55E−06
hsa-miR-200c	Down	−2.4379	1.58E−06
hsa-miR-486-5p	Down	−2.65039	0.006190634
hsa-miR-203	Down	−2.84448	0.002550727
hsa-miR-200b	Down	−2.91643	1.71E−04
hsa-miR-338-3p	Down	−2.94236	0.011949077
hsa-miR-429	Down	−3.37992	0.010056388
hsa-miR-224*	Down	−4.27994	0.006564327

**Table 2:** The 10 most upregulated and six most downregulated microRNAs differentially expressed, based on microarray, in the different forms (TT + BT + BB + BL + LL) vs. healthy control, fold change (FC) ≥ |2| and *p* ≤ 0.05.

Gene symbol	Regulation	FC microarray	*p* Value
hsa-miR-155	Up	16.85744	3.84E−20
hsa-miR-146a	Up	7.594823	8.38E−14
hsa-miR-142-5p	Up	6.288545	1.96E−13
hsa-miR-21*	Up	5.760914	8.59E−08
hsa-miR-142-3p	Up	5.004883	7.57E−11
hsa-miR-21	Up	4.018055	6.73E−12
hsa-miR-146b-5p	Up	3.947622	4.07E−06
hsa-miR-150	Up	3.921194	6.15E−09
hsa-miR-181a*	Up	3.792876	9.06E−09
hsa-miR-342-5p	Up	3.128596	4.68E−09
hsa-miR-429	Down	−2.14509	−2.145087
hsa-miR-141	Down	−2.17806	−2.1780634
hsa-miR-205	Down	−2.21269	−2.2126882
hsa-miR-193b	Down	−2.22064	−2.2206442
hsa-miR-200c	Down	−2.26256	−2.2625608
hsa-miR-224*	Down	−2.26869	−2.2686923

In relation to reactional status, we observed that 37 miRNAs were differentially expressed in reactional type 1 samples (Tables S10 and S12 in Supplementary Material). The 10 most upregulated or downregulated miRNAs differentially expressed in type 1 reaction and HC are shown in Table 3. In the comparison between R1 vs. R1 respective clinical forms, the hsa-miRNA-378* is exclusive. Regarding type 2 reactions, 26 miRNAs were differentially expressed in these samples (Tables S11 and S13 in Supplementary Material). The 10 most upregulated and 7 downregulated miRNAs differentially expressed in type 2 reaction and HC are shown in Table 4. When comparing type 2 reaction (R2) samples with their respective clinical forms (BB + BL), one miRNA (hsa-miR-20a*) was differentially expressed (upregulated) (Table S11 in Supplementary Material).

**Table 3:** The 10 most upregulated or downregulated microRNAs differentially expressed in reaction type 1 (R1) vs. healthy control, fold change (FC) ≥ |2| and *p* ≤ 0.05.

Gene symbol	Regulation	FC microarray	*p* Value
hsa-miR-155	Up	21.27233	5.77E−12
hsa-miR-21*	Up	11.91278	8.96E−11
hsa-miR-146a	Up	9.099005	2.64E−08
hsa-miR-142-5p	Up	8.484986	1.66E−09^a^
hsa-miR-150	Up	5.275174	6.62E−09
hsa-miR-181a*	Up	5.255039	1.32E−08
hsa-miR-142-3p	Up	5.105462	5.94E−06^a^
hsa-miR-21	Up	4.870885	7.12E−09
hsa-miR-146b-5p	Up	4.820066	2.46E−04^a^
hsa-miR-342-5p	Up	4.810751	1.59E−10
hsa-miR-133b	Down	−3.04698	5.46E−06
hsa-miR-205	Down	−3.06662	1.09E−07
hsa-miR-1290	Down	−3.24258	1.07E−04^a^
hsa-miR-224*	Down	−3.48345	7.73E−06
hsa-miR-224	Down	−3.75265	7.50E−05
hsa-miR-200b	Down	−4.04193	5.27E−07
hsa-miR-200a	Down	−4.24384	8.55E−06
hsa-miR-429	Down	−4.24633	5.53E−08
hsa-miR-96	Down	−5.11292	2.55E−05
hsa-miR-203	Down	−5.17564	6.86E−05

*^a^Validated miRNA*.

**Table 4:** The 10 most upregulated and seven most downregulated microRNAs differentially expressed in reaction type 2 (R2) vs. healthy control, fold change (FC) ≥ |2| and *p* ≤ 0.05.

Gene symbol	Regulation	FC microarray	*p* Value
hsa-miR-21*	Up	19.92985	3.25E−10
hsa-miR-155	Up	9.499085	1.63E−07
hsa-miR-223	Up	8.636663	2.38E−04
hsa-miR-142-5p	Up	7.731227	5.34E−08^a^
hsa-miR-142-3p	Up	5.971354	1.98E−04^a^
hsa-miR-146b-5p	Up	5.863311	2.11E−04^a^
hsa-miR-21	Up	5.585104	2.01E−09
hsa-miR-150	Up	5.319824	1.94E−05
hsa-miR-181a*	Up	5.028419	3.16E−07
hsa-miR-7	Up	4.940581	1.29E−05
hsa-miR-214	Down	−2.14012	1.71E−04
hsa-miR-125b-2*	Down	−2.17508	7.85E−05
hsa-let-7b	Down	−2.40185	2.46E−05
hsa-miR-193b	Down	−2.41444	1.08E−05
hsa-miR-205	Down	−2.54304	2.15E−04
hsa-miR-429	Down	−2.64966	2.54E−04
hsa-miR-200c	Down	−2.68127	7.31E−06

*^a^Validated miRNA*.

In general, miRNAs were heterogeneously expressed. The majority of these miRNAs were differentially expressed in different disease forms or reactional states compared to expression in the HC group, but with higher or lower magnitude (hsa-miR-142-5p, hsa-miR-155-5p, hsa-miR-181a*, and hsa-miR-21-3p, among others). However, some were expressed only in specific groups (type 1 reactions: hsa-miR-1290, hsa-miR-200a, hsa-miR-200b, hsa-miR-205*, hsa-miR-34a, hsa-miR-501-3p, hsa-miR-27a, hsa-miR-27b, hsa-miR-133b, hsa-miR-224, hsa-miR-96, hsa-miR-203, hsa-miR-378, and hsa-miR-500a*; type 2 reactions: hsa-miR-125b-2*, hsa-miR-214, hsa-miR-7, hsa-miR-629, hsa-miR-20a*, and hsa-miR-223).

Of the 51 miRNAs subjected to RT-PCR validation, 8 were validated (hsa-miR-1290, hsa-miR-142-3p, hsa-miR-142-5p, hsa-miR-146b-5p, hsa-miR-342-3p, hsa-miR-361-3p, hsa-miR-3653, and hsa-miR-484). All miRNAs subjected to validation and the associated values for the comparisons are shown in Table 5.

**Table 5:** Results of microRNA validation with respective target genes comparison.

Symbol	Real time	Microarray	*p* Value	Regulation	Validated form	Target gene	miRBase
hsa-miR-1290	−6.418556575	−3.2425804	0.0319	Down	R1 vs. healthy control (HC)		MIMAT0005880
hsa-miR-142-3p	72.40788202	5.157617	0.0001	Up	Disease vs. HC	AKR1B10	MIMAT0000434
hsa-miR-142-3p	46.32763259	5.105462	0.0248	Up	R1 vs. HC		MIMAT0000434
hsa-miR-142-3p	50.73379106	5.9713535	0.0046	Up	R2 vs. HC		MIMAT0000434
hsa-miR-142-5p	13.50652073	6.894877	0.0001	Up	Disease vs. HC		MIMAT0000433
hsa-miR-142-5p	11.24670109	8.484986	0.0095	Up	R1 vs. HC		MIMAT0000433
hsa-miR-142-5p	7.586663505	7.731227	0.0198	Up	R2 vs. HC		MIMAT0000433
hsa-miR-146b-5p	7.640099529	4.3596835	0.0001	Up	Disease vs. HC		MIMAT0002809
hsa-miR-146b-5p	7.006850053	4.8200655	0.0030	Up	R1 vs. HC		MIMAT0002809
hsa-miR-146b-5p	8.340527806	5.8633113	0.0002	Up	R2 vs. HC		MIMAT0002809
hsa-miR-342-3p	6.380637592	2.780538	0.0001	Up	Disease vs. HC	FAM180B	MIMAT0000753
hsa-miR-361-3p	5.662851322	2.0504081	0.0001	Up	Disease vs. HC		MIMAT0004682
hsa-miR-361-3p	6.351012385	2.0587645	0.0185	Up	R2 vs. HC		MIMAT0004682
hsa-miR-3653	2.298881007	2.0732837	0.0122	Up	Disease vs. HC		MIMAT0018073
hsa-miR-484	2.195647906	2.6891153	0.0056	Up	R2 vs. HC	FASN	MIMAT0002174

*Disease = (TT + BT + BB + BL + LL + R1 + R2)*.

### miRNA/mRNA Target Pairs Opposite Expression Pattern

MicroRNA/mRNAs found as differentially expressed in each of the following group comparisons (Clinical forms vs. HC, TT vs. HC, LL vs. HC, R1 vs. HC and R2 vs. HC) are detailed in Table S14 in Supplementary Material. In summary, in the comparison Clinical forms vs. HC, six miRNAs (hsa-miRNA-429, hsa-miRNA-142-3p, hsa-miRNA-142-5p, hsa-miRNA-146b, hsa-miRNA-342-3p, and hsa-miRNA-342-5p) are associated with 39 mRNAs. In the comparison TT vs. HC, 6 miRNAs (hsa-miRNA-429, hsa-miRNA-142-3p, hsa-miRNA-142-5p, hsa-miRNA-342-3p, hsa-miRNA-342-5p, and hsa-miRNA-361-3p) are associated with 27 mRNAs. In the comparison LL vs. HC, 4 miRNAs (hsa-miRNA-429, hsa-miRNA-142-5p, hsa-miRNA-342-3p, and hsa-miRNA-484) are associated with 44 mRNAs. In the comparison R1 vs. HC, 9 miRNAs (hsa-miRNA-429, hsa-miRNA-133b, hsa-miRNA-142-3p, hsa-miRNA-142-5p, hsa-miRNA-146b-5p, hsa-miRNA-342-3p, hsa-miRNA-342-5p, hsa-miRNA-361-3p, and hsa-miRNA-501-3p) are associated with 62 mRNAs. In the comparison R2 vs. HC, 6 miRNAs (hsa-miRNA-429, hsa-miRNA-142-3p, hsa-miRNA-142-5p, hsa-miRNA-146b-5p, hsa-miRNA-361-3p, and hsa-miRNA-484) are associated with 57 mRNAs. After comparing the miRNAs validated in this study with the mRNAs validated by Belone et al.’s study, the following mi/mRNAs were identified: hsa-miRNA-142-3p and AKR1B10, hsa-miRNA-342-3p and FAM180b, and hsa-miRNA-484 and FASN (Table 5).

### Pathway Analysis

The pathways obtained by the different comparisons are detailed in Table S16 in Supplementary Material. The following signaling pathways were obtained by the comparisons: fatty Acyl-CoA Biosynthesis, ChREBP activates metabolic gene expression, Triglyceride Biosynthesis, metabolism of water-soluble vitamins and cofactors, Gap junction trafficking, fatty Acyl-CoA biosynthesis, Gap junction trafficking and regulation, ChREBP activates metabolic gene expression, activation of gene expression by SREBF (SREBP), regulation of cholesterol biosynthesis by SREBP (SREBF), synthesis of very long-chain fatty acyl-CoAs, linoleic acid metabolism.

## Discussion

Analysis of the human genome has indicated that many genomic sequences encode RNA that does not encode protein. Many longer non-coding RNAs, small nucleolar RNAs, miRNAs, and other small regulatory RNAs are included among the non-coding RNAs. miRNAs (20–24 nucleotides) have been extensively studied. They consist of a group of small non-coding RNA molecules related to small interfering RNAs. miRNAs play important roles in key biological processes such as gene regulation, cell growth, apoptosis, and hematopoietic lineage differentiation. As such, miRNAs are involved in various human diseases such as cancer, vascular disease, immune disease, and infections (17). Regarding neoplasms, it is clear that miRNAs are differentially expressed between normal and cancerous cells, that they reflect tissue-specific expression signatures, and that they can either promote (“oncomiRs”) or suppress tumor development and progression, thereby influencing all of the hallmarks of cancer. Many miRNAs have been used for early detection, diagnosis, and prognosis in different types of cancer (9, 18). miRNAs are also deregulated in several non-neoplastic diseases. There are many publications showing that they play an important role in the dysregulation of the immune response in inflammatory/autoimmune diseases (such as systemic lupus erythematous, rheumatoid arthritis, and multiple sclerosis) and infectious diseases (including tuberculosis and leprosy).

In this study, a large number of differentially expressed miRNAs were identified mainly due to the varied and polymorphous composition of the cells that contributed to the inflammatory processes in leprosy skin lesions (4, 5, 14). In the tuberculoid forms (TT and BT), bacilli are absent or rarely found in neural branches, macrophages, or mononuclear cells of the papillary dermis. In contrast, in the lepromatous forms (BL and LL), bacilli are abundant and can parasitize virtually all tissues. Therefore, it is likely that these differentially expressed miRNAs are involved in bacillary proliferation and dissemination.

Many miRNAs described in the literature are associated with the regulation of the immune response in other diseases; for example, hsa-miR-34, hsa-miR-142-3p, hsa-miR-146a, hsa-miR-150, hsa-miR-155, hsa-miR-214, hsa-miR-223, and hsa-miR-424 were also present in leprosy tissues (Tables S2, S3, and S13 in Supplementary Material; Table 5). Recently, several miRNAs were described as being important in neural diseases. Leprosy, due to the tropism of *M. leprae* for peripheral neural branches, is a predominantly neural infectious disease. Initially, in its indeterminate phase, before the development of lesions within the R&J spectrum, the peripheral neural branches of the skin or subcutaneous tissue are involved, and this is characterized by minimal inflammatory infiltration, which is predominantly lymphocytic, without well-formed granulomas. Subsequently, with complete disease establishment, granulomas involving neural branches become a histological feature present in all forms and both reactional states of leprosy. Therefore, there is constant interaction between neural branches and the inflammatory process during the disease.

Some studies showed that miRNAs play important roles in the development of mycobacterial diseases (tuberculosis, leprosy, and *Mycobacterium avium* infection), probably by regulation of the immune response of the hosts. Functional profiles and experiments generated evidence suggesting that regulation of specific miRNAs during infection might stimulate the immune response or facilitate immune evasion by the pathogens (19, 20). They have also been involved in the regulation of the host immune response in relation to other bacteria such as *Salmonella, Helicobacter pylori, Francisella tularensis*, and *Listeria monocytogenes* (21). miRNA17 participates in the regulation of autophagy in macrophages in tuberculosis. *Mycobacterium tuberculosis* (Mtb) infection leads to the downregulation of miRNA17 and consequently the upregulation of its targets Mcl-1 and STAT3 (22). In addition, miRNA expressed in Mtb-infected macrophages revealed the downregulation of miR-let-7f, which was dependent on the Mtb-secreted effector ESAT-6 (23). It was shown that let-7f targets A20, a feedback inhibitor of the NF-κB pathway. Experimental studies in mice infected with Mtb showed decrease in let-7f expression and increase in A20 during progression of the infection. A20-deficient macrophages result in decreased Mtb survival. Moreover, production of tumor necrosis factor (TNF), interleukin (IL-1β), and nitrites, which are mediators of immunity to Mtb, is consequently increased. Furthermore, the overexpression of let-7f diminishes Mtb survival and augments the production of the TNF and IL-1β cytokines. These results suggest let-7f and its target A20 play a role in regulation of the immune response against Mtb and control of mycobacterial burden (23). To ensure their survival and replication, bacterial pathogens manipulate a wide range of host cell functions by providing effector proteins to host cells. Regulation of miRNA expression by bacterial pathogens is emerging as an essential part of the host’s response to infection, as is the discovery of molecular mechanisms exploited by bacteria to control the microenvironment of host cells (11).

There have been a few studies on the expression of miRNAs specifically related to leprosy. Liu et al. identified 13 miRNAs that were differentially expressed in the lesions of subjects with LL in comparison to expression in the self-limiting TT disease. Bioinformatics analysis revealed marked enrichment of LL-specific miRNAs, which target key immune genes shown to be downregulated in LL tissue compared to TT lesions. The most differentially expressed miRNA in LL lesions, hsa-mir-21, was upregulated in *M. leprae*-infected monocytes. hsa-mir-21 inhibited gene expression of vitamin D-dependent antimicrobial peptides, CAMP and defensin Beta 4A, through downregulation of toll-like receptor 2/1 (TLR2/1)-induced cytochrome p450 27B1 and IL-1β, and upregulation of IL-10. Thus, the ability of *M. leprae* to upregulate hsa-mir-21 could result in the regulation of multiple genes associated with the LL disease form, providing an effective mechanism to escape from vitamin D-mediated antimicrobial pathway (24). Our results show that miRNA21 and miRNA21* are upregulated both in the leprosy spectrum and in the reactional states (Tables S1, S2, S13, and S14 in Supplementary Material). Although we did not find significant differences in terms of miRNA 21/miRNA21* expression between TT and LL, there were significant similarities with the work of Liu et al. We observed that the expression of miRNA21/miRNA21* was higher in lepromatous form samples and type 2 reactions [LL vs. HC; (BL + LL) vs. HC and R2 vs. HC] when compared to that in the tuberculoid forms and type 1 reactions [TT vs. HC; (TT + BT) vs. HC and R1 vs. HC] (Tables S4–S7, S12, and S13 in Supplementary Material).

Jorge et al. recently published a study on the expression of miRNAs in leprosy polar forms. After evaluating the expression of 377 miRNAs by TaqMan Low Density Array (TDLA) in skin samples of patients with the polar leprosy forms (TT and LL) and HC, the authors identified four validated miRNA (hsa-miR-101, hsa-miR-196b, hsa-miR-27b, and hsa-miR-29c) that can be used to discriminate HC from leprosy patients with 80% sensitivity and 91% specificity, respectively. In addition, the same miRNAs can discriminate, with 83% sensitivity and 80% specificity, LL from TT patients (25). In this study, one miRNA (hsa-miR-181a*) was found differentially expressed in the comparison TT vs. LL (Table S4 in Supplementary Material). None of the miRNAs validated by Jorge et al. were identified in this study when comparing TT vs. HC (Table S7 in Supplementary Material) and LL vs. HC (Table S8 in Supplementary Material); however, hsa-miR-27b was upregulated in R1 reaction (Table S12 in Supplementary Material). Cezar-de-Mello et al. reported that pre-miR-146a, which is known to modulate TNF levels, exhibits polymorphisms that are associated with susceptibility to leprosy. In this study, which was performed on skin samples, although we observed increased levels of miR-146a, it was not possible to correlate this with any specific form of leprosy (26). We observed that miR-146a is upregulated in all forms of leprosy and exhibits higher expression in the lepromatous form compared to that in the tuberculoid form (Tables S2, S4–S6, and S7 in Supplementary Material). There were also no significant differences when expression values were compared between the forms. Between the two types of reactions, only R1 presented with significantly different expression compared to that of HC samples (Table S13 in Supplementary Material). Regarding inflammatory/infectious diseases, hsa-miR-146a is downregulated in peripheral blood mononuclear cells at different stages of chronic hepatitis B virus infection (27). The miRNA hsa-miR-146a is crucial for the progression of Alzheimer’s disease and functions through the hsa-miR-146a/STAT1/MYC pathway (28). In Kaposi’s sarcoma (KS), this miRNA is upregulated and is associated with the downregulation of CXCR4; this might contribute to the development of KS by promoting the premature release of KS-associated herpes virus-infected endothelial progenitors into the circulation (29).

A study by Kumar et al. showed that immunological dysregulation might lead to hyporeactivity or anergy in T cells in *M. leprae*-infected patients. It also identified Cbl-b overexpression and loss of miR-181 expression as important characteristics for the progression of leprosy (30). In this study, we determined that miR-181a* is upregulated throughout the disease spectrum and reactional states, indicating that it takes part in the pathophysiological processes of the disease. However, we found higher expression in the lepromatous forms (LL) than in the tuberculoid forms (TT) (Tables S3, S6, and S7 in Supplementary Material). The regulation of T cell sensitivity by miR-181a allows mature T cells to recognize antagonists (inhibitory peptide antigens) as agonists. These effects may be achieved by the downregulation of multiple phosphatases, which results in elevated steady-state levels of phosphorylated intermediates and reduction in the signaling threshold of T cell receptor. It is important to mention that higher miR-181a expression correlates with greater T cell sensitivity in immature T cells. This suggests that miR-181a acts as an intrinsic antigen sensitivity “rheostat” during development of T cells (31).

Another very relevant aspect of leprosy is its reactional episodes. However, there were no previous references in the literature regarding miRNA expression in leprosy reactions. In respect to type 1 reactions, we observed that 14 miRNAs were differentially expressed only in these samples. Several of these miRNAs are described in the literature related to diseases such as lung, breast, and kidney cancer (32–34). Upregulation of hsa-miR-34a and hsa-miR-500a is associated with the development of neuroblastoma and is related to poor response to chemotherapy in non-small cell lung carcinoma (NSCLC) (35, 36). In the comparison R1 vs. R1 respective clinical forms (Table S9 in Supplementary Material), the miRNA-378* is exclusive. The literature shows that miRNA-378* is associated with lipids metabolism (37).

Regarding type 2 reactions, six miRNAs were differentially expressed exclusively in these samples. Based on the literature, downregulation of hsa-miR-125b-2* and hsa-miR-214 is associated with miscarriages, development of gastric adenocarcinoma in the elderly (compared to that in young individuals), glioma cell proliferation, and germ cell tumor growth in the testis (34, 38, 39). In addition, the upregulation of hsa-miR-223 is directly related to periodontitis and gastric cancer (40, 41). It is not currently known how these miRNAs participate in the initiation and/or maintenance of type 1 and type 2 reactions. Clinical and histopathological characteristics are different between reactions and clinical forms. There are also important changes that occur in the composition of granulomas, in the phenotype of interstitial cells, and in angiogenesis between these cell types. Thus, the clinical and histopathological characteristics specific to the various forms and reactions of the disease could be explained by differentially expressed miRNAs. The role of these miRNAs in leprosy is unknown.

Of the eight miRNAs validated by RT-PCR in this study (hsa-miR-1290, hsa-miR-142-3p, hsa-miR-142-5p, hsa-miR-146b-5p, hsa-miR-342-3p, hsa-miR-361-3p, hsa-miR-3653, and hsa-miR-484), there are no references in the literature regarding their expression in leprosy skin lesions. hsa-miR-1290, validated as downregulated in type 1 reactions compared to that in HCs, is associated with the suppression of proliferation and invasion in NSCLC and is significantly downregulated in luminal-A breast tumors (42). Its potential target, arylamine *N*-acetyltransferase 1, is correlated with increased survival in patients with these tumor subtypes (33). hsa-miR-139-5p, which was downregulated in disease samples compared to expression in HC tissues, plays a pivotal role in lung cancer and breast cancer. It inhibits cell proliferation and metastasis and promotes apoptosis by targeting oncogenic c-Met (43, 44). hsa-miR-142-3p, which was upregulated in disease samples vs. HC, R1 vs. HC, and R2 vs. HC, is associated with bromocriptine-resistant prolactinoma and is present in inflamed gingival tissue but not in healthy gingival tissue (45, 46). hsa-miR-142-5p, which was upregulated in disease vs. HC, R1 vs. HC, and R2 vs. HC, in combination with hsa-miR-375, was reported to be a predictor of disease progression, showing potential to predict recurrent gastric cancer; hsa-miR-142-5p is involved in the regulation of several oncogenic signaling pathways such as vascular endothelial growth factor hsa-miR-142-5p, TP53, MAPK, and Wnt (47). hsa-miR-146b-5p, which was upregulated in disease vs. HC, R1 vs. HC, and R2 vs. HC, is also upregulated in recurrent glioblastoma compared to expression in primary glioblastoma, suggesting that it might be related to disease relapse (48). hsa-miR-342-3p, which was found to be upregulated in disease vs. HC and R1 vs. HC, might play a general role in late stage prion disease and this way be used as a marker for animal and human spongiform encephalopathies (49). hsa-miR-361-3p, which was found to be upregulated in disease vs. HC and R2 vs. HC, is upregulated in the inflamed mucosa of Crohn’s disease patients compared to expression in non-inflamed mucosa (50). The function of hsa-miR-3653, found to be upregulated in disease vs. HC, is virtually unknown. hsa-miR-484, which was upregulated in R2 vs. HC, is associated with chemoresistance in ovarian cancer due to enhanced angiogenesis, resulting from modulation of the tumor vasculature, through regulation of VEGFB and VEGFR2 pathways (51). Interestingly, increased angiogenesis occurs in skin lesions in the entire spectrum of leprosy and in both reactional states compared to that in HCs; the number of vessels was observed to be higher in R2 tissues than in any of the other disease forms or the R1 stage (52).

MicroRNAs are increasingly described as important players in the regulation of cellular functions. However, the differential expression of miRNAs and target mRNAs in tissues, fluids, or cultured cells does not necessarily mean that there is a related disorder in cell metabolism. Most miRNAs were validated only experimentally with studies directed at the target mRNA. As a single miRNA species can regulate hundreds of mRNAs and because several miRNAs can be regulated by a single mRNA, the broad analysis of multiple mRNA/miRNA interactions becomes very complex, and often these interactions remain unknown as a result. Recently, we published a study on the expression of mRNAs in leprosy using the same samples and types of comparisons used in this study (12). Comparing the mi/mRNAs present in both studies, we observed several miRNAs opposed to mRNAs with different functions. The signaling pathways obtained indicate predominant changes in the lipid metabolism, cellular trafficking, and metabolism of soluble vitamins and its cofactors. They have been associated with different diseases and immunological processes in the literature (53–57).

In summary, this study has uncovered miRNAs that are deferentially expressed in leprosy skin lesions, several of which were described for the first time in this disease. miRNA expression profiles as they relate to the spectrum of leprosy and the reactional states might provide a solid basis for the understanding of the pathophysiological mechanisms of leprosy. These miRNAs will require validation and functional analyses to evaluate their role in the pathogenesis of leprosy. Thus, this study represents the initial stages of an important field of research to identify molecular markers of this disease or its reactional states. Furthermore, these deregulated miRNAs and their respective signaling pathways could be used as therapeutic targets, consequently enabling the development of novel drugs for the treatment of leprosy.

## Author Contributions

CS, AB, DC, and FS conceived the project. CG and CS performed clinical evaluation of patients and performed biopsy procedures. CS performed histopathological and immunohistochemical analysis. AB, AC, MP, LF, and AT were involved with RNA sample preparation, hybridization, and PCR. CS, MP, AC, AB, AT, LF, RR, and PR were responsible for data analysis. CS and PR wrote the manuscript. All the authors critically revised the paper for intellectual content.

## Conflict of Interest Statement

The authors declare that the research was conducted in the absence of any commercial or financial relationships that could be construed as potential conflict of interest.

## Footnotes

^1^http://www.cytoscape.org/.

^2^http://www.reactome.org.

^3^http://www.ncbi.nlm.nih.gov/geo/.
